# A nuclear Pandora’s box: functions of nuclear envelope proteins in cell division

**DOI:** 10.1093/aobpla/plac065

**Published:** 2022-12-25

**Authors:** M Arif Ashraf

**Affiliations:** Department of Biology, University of Massachusetts Amherst, Amherst, MA 01003, USA

**Keywords:** Cell division, land plant evolution, LINC complexes, mitosis, nuclear envelope proteins, nuclear pore complex

## Abstract

The nucleus is characteristic of eukaryotic cells and nuclear envelope proteins are conserved across the kingdoms. Over the years, the function of these proteins was studied in the intact nuclear envelope. Knowledge regarding the localization and function of nuclear envelope proteins during mitosis, after the nuclear envelope breaks down, is limited. Until recently, the localization of nuclear envelope proteins during mitosis has been observed with the mitotic apparatus. In this context, research in plant cell biology is more advanced compared to non-plant model systems. Although current studies shed light on the localization of nuclear envelope proteins, further experiments are required to determine what, if any, functional role different nuclear envelope proteins play during mitosis. This review will highlight our current knowledge about the role of nuclear envelope proteins and point out the unanswered questions as future direction.

## Teaser

During open mitosis, the nuclear envelope breaks down. As a result, the nuclear membrane proteins are not intact in the envelope during cell division. The question is, where do these nuclear membrane proteins localize during cell division? In this review article, localization of nuclear membrane proteins during mitosis is highlighted. At the same time, the functional role of nuclear membrane proteins in cell division is discussed. In this context, the evolution of nuclear membrane proteins in land plant species provides unique opportunities for future research.

## Introduction

**Figure 1.**
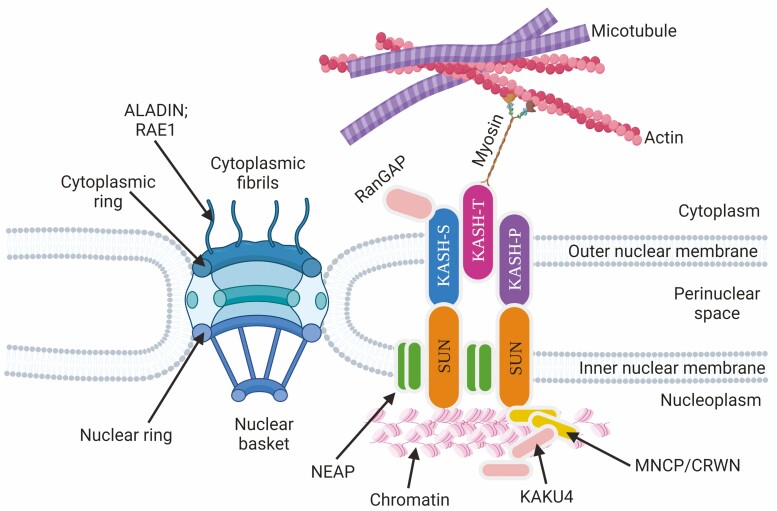
Structure and organization of nuclear membrane and pore complex proteins. The outer and inner nuclear membranes consist of KASH and SUN domain, respectively, containing proteins. KASH domain-containing proteins are categorized into subclass as S, T (interact with actin through myosin) and P type. Other nuclear membrane-associated proteins, such as NEAP, MNCP/CRWN and KAKAU4, were highlighted as well. Nuclear pore complex perforates double nuclear membranes and facilitates import and export of molecules. Nuclear pore complex proteins, such as ALADIN and RAE1, localize at the cytoplasmic fibrils.

Eukaryotic cells contain compartmentalized organelles, such as nuclei. The nucleus is fundamentally important for protecting genetic materials. The nuclear membrane is double-layered and therefore has an outer and inner nuclear membrane. Similar to any other membrane, both outer and inner nuclear membranes have embedded proteins. Due to their spatial localizations, nuclear membrane proteins interact and perform distinct functions. For instance, the inner nuclear membrane proteins interact with chromatin and nuclear lamins. The outer nuclear membrane proteins interact with the cytoskeleton and motor proteins (Starr 2019) (Fig. 1). Both the outer and inner nuclear membranes are perforated by the nuclear pore complex (NPC). The NPC acts as a channel to facilitate the import and export of proteins and RNAs between the nucleus and the cytoplasm (Jiang 2022) (Fig. 1).

The nuclear membrane and NPC proteins play crucial roles for maintaining nuclear morphology, movement, the import and export of molecules, mechanosensing, regulation of gene expression, spatial chromatin organization and cell division. The nucleus was the first organelle discovered by Robert Brown after the cell itself because it is clearly visible under the microscope. As a result, the shape and size of the nucleus were studied extensively for a long time across the eukaryotic kingdom (Mukherjee *et al.* 2016). Due to the improvement of microscopy techniques, such as video microscopy, observations of nuclear movement and its dynamics within the cell were observed in greater detail (Gundersen and Worman 2013). Additionally, fluorescent tagging of proteins helped us to discover that NPC proteins facilitate import and export of molecules between the nucleus and cytoplasm (Kim *et al.* 2017). Furthermore, nuclear envelope proteins reside in both the outer and inner membrane and act as a bridge to transfer signals from the cell cortex to regulate gene expression (Cho *et al.* 2017). This example emphasizes the role of nuclear membrane proteins as mechanosensors. These mentioned examples are based on the functional roles of nuclear envelope proteins while the envelope remains intact. But the function of nuclear envelope proteins between the envelope breakdown and reappearance in the daughter cells during cell division is not observed in detail.

The above-mentioned functions are directly and indirectly mediated by two major classes of nuclear envelope proteins: nuclear membrane proteins and NPC proteins. Among them, the well-studied LINC (Linker of Nucleoskeleton and Cytoskeleton) complex maintains the link between the nucleus and the cytoskeleton. The KASH domain of outer nuclear membrane proteins interacts with the cytoskeleton. The SUN domain contains inner nuclear membrane proteins and interacts with chromatin and lamins. At the same time, the outer nuclear membrane proteins interact with the inner nuclear membrane proteins via the KASH and SUN domains, respectively (Starr 2019). Apart from LINC complexes or nuclear membrane-localized proteins, the other major group of proteins belongs to NPC. There is a major progress in understanding the human NPC at the atomic level. The human NPC contains ~1000 proteins, including 30 nucleoporins (NUPs), where NUPS are the major proteins of NPC (Jiang 2022). Nuclear pore complexes act as a channel embedded in the nuclear envelope and perform various functions. As a result, NPC and associated proteins are a major focus for diseases in humans.

**Figure 2.**
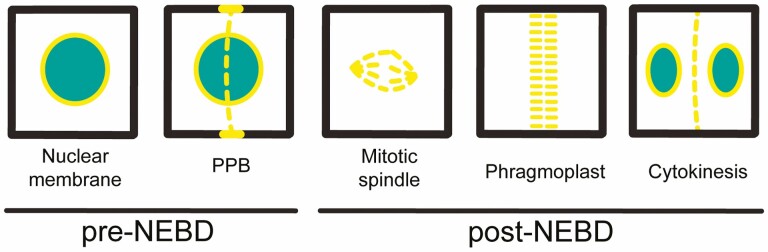
Localization of nuclear envelope proteins in pre- and post-NEBD. Expected and universal localization of nuclear envelope proteins before cell division and after cell division. Other possible localization of nuclear envelope proteins including PPB, spindle, phragmoplast and new cell plate.

This review will focus on nuclear envelope proteins in plants. Recent review articles comprehensively covered the progress and discovery in non-plant systems (Koch and Yu 2019; Lin and Hoelz 2019; Tingey *et al.* 2019; Pawar and Kutay 2021). While animal and yeast nuclear envelope proteins have received much attention, comparatively little is known about their homologues in plants. Interestingly, one of the major areas regarding nuclear envelope proteins’ function, determination of future division site and during mitosis, is advanced in the plant system compared to non-plant model organisms. In this review, the involvement of nuclear envelope proteins in future division site determination is explored along with the possible role they play during mitosis will be explored extensively and will provide the direction to future research for unanswered questions (Fig. 2).

## Nuclear Protein Localization After the Envelope Breakdown

**Figure 3.**
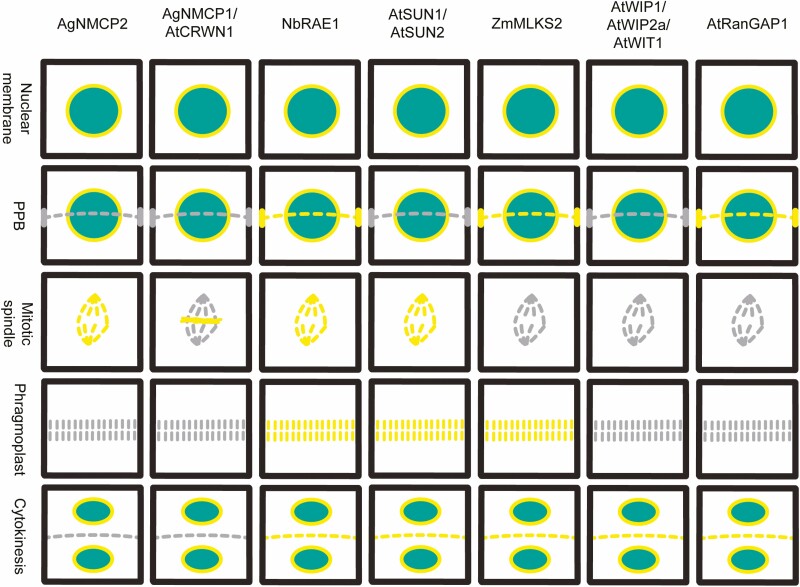
Localization of nuclear envelope proteins during cell division. The localization of nuclear envelope proteins (AtRanGAP1, AtWIP1, AtWIP2a, AtWIT1, ZmMLKS2, AtSUN1, AtSUN2, NbRAE1, AgNMCP1, AgNMCP2, AtCRWN1) during interphase (nuclear envelope and PPB), mitosis (spindle, phragmoplast, cytokinesis).

RanGAP is one of the most studied nuclear membrane proteins for its localization during mitosis. It contains the plant-specific motif Tryptophan (W)–Proline (P)–Proline (P), or in short WPP motif (Rose and Meier 2001). This WPP domain of AtRanGAP1 ensures nuclear membrane targeting in the interphase stage (Rose and Meier 2001). Interestingly, in *Arabidopsis*, RanGAP1 localizes at the preprophase band (PPB), cortical division site (CDS), kinetochore region and new cell plate. This cellular observation was further confirmed in a co-localization study with microtubule marker (Xu *et al.* 2008). It has been found that the WPP domain of AtRanGAP1 not only facilitates nuclear envelope targeting, but also localization to the PPB, CDS, kinetochore and new cell plate (Fig. 3). Even after testing with microtubule-depolymerizing drug oryzalin, PPB-specific localization of AtRanGAP1 persists, which indicates a microtubule-independent mechanism (Xu *et al.* 2008). In the PPB-depleted mutant *ton2-14*, RanGAP1 is abolished from the PPB and CDS (Xu *et al.* 2008). The observation suggests the possibility of FASS/TONNEAU2-mediated targeting of AtRanGAP1 in the PPB.

WPP was the first identified nuclear envelope-targeting domain in plants (Rose and Meier 2001). The WPP domain was utilized to identify other nuclear membrane proteins in plants, including AtWIP1, AtWIP2a, AtWIP2b, AtWIP3, AtWIT1 and AtWIT2 (Xu *et al.* 2007; Zhao *et al.* 2008). WIP/WIT protein localization is observed in the nuclear membrane, as expected. A subset of these proteins (AtWIP1, AtWIP2a, AtWIT1) are also localized in the new cell plate during cytokinesis (Xu *et al.* 2007; Zhao *et al.* 2008) (Fig. 3). Although these outer nuclear membrane proteins have indistinguishable interphase localization, their localization is distinctive during mitosis. This highlights the contrasting mechanism of cell plate targeting during cytokinesis although the functional relevance is unknown. AtRanGAP1 localization in the triple (*wip1-1 wip2-1 wip3-1*) and double (*wit1-1 wit2-1*) mutant background corroborates with the distinct cell plate targeting hypothesis. Because, in the *wip1-1 wip2-1 wip3-1* and *wit1-1 wit2-1* background, AtRanGAP1 signal is abolished from the nuclear envelope, but remains same in the new cell plate (Xu *et al.* 2007; Zhao *et al.* 2008).

Stable transformation of *Arabidopsis* inner nuclear membrane proteins, AtSUN1 and AtSUN2, with the chromatin marker histone in tobacco BY-2 cells provided the opportunity to observe the localization of inner nuclear membrane proteins in post-nuclear envelope breakdown (NEBD). As expected, based on the characteristics of these two inner nuclear membrane proteins, both AtSUN1 and AtSUN2 localize (using constitutive 35S promoter) in the nuclear envelope during interphase and prophase. After the envelope breakdown, they were first observed around the mitotic spindle area during metaphase. Afterwards, both AtSUN1 and AtSUN2 are observed around the phragmoplast and new cell plate area (Fig. 3). The formation of two daughter cells leads to the reappearance of AtSUN1 and AtSUN2 on the nuclear envelope (Graumann and Evans 2011). These observations suggest that the inner nuclear membrane proteins localize around the mitotic spindle, phragmoplast and new cell plate after the NEBD, but the co-localization with microtubules was not examined in this study.

Almost at the same time, another report was published regarding the localization of nuclear membrane proteins after the NEBD, where AtSUN1 was expressed together with microtubule (GFP-TUB6), chromosome (AtHTB1-GFP/CENH3–GFP) and endoplasmic reticulum (GFP–HDEL) markers, in *Arabidopsis* suspension-cultured cells. Co-expressing AtSUN1 and microtubule demonstrated that at the interphase and prophase stage, after envelope breakdown, AtSUN1 localizes around the mitotic spindle forming region. As the mitotic spindle shortens and separates the chromosomes, AtSUN1 accumulates preferentially distal to the future division site. Interestingly, localization was not observed at the phragmoplast, but localization at the new cell plate was clearly observed (Oda and Fukuda 2011). Both studies highlight a similar localization pattern of inner nuclear AtSUN1 in tobacco BY-2 and *Arabidopsis* suspension culture cells during mitosis. Another class of nuclear membrane protein, NEAPs (nuclear envelope-associated proteins), interact with inner nuclear membrane protein SUN (Gumber *et al.* 2019a). But, the localization of NEAPs during the mitosis has not been reported yet.

Additional localization examples for nuclear envelope proteins during mitosis appeared recently. *Zea mays* outer nuclear membrane protein, MLKS2 (Maize LINC KASH AtSINE-like 2), is involved in proper nuclear positioning during asymmetric cell division. Mispositioning of nucleus in *mlks2* mutant leads to misplacement of the PPB and eventually leads to misoriented asymmetric cell division (Ashraf *et al.* 2022). Heterologous overexpression of ZmMLKS2 in tobacco pavement cells demonstrates nuclear envelope-specific localization during interphase and prophase (Gumber *et al.* 2019b; Ashraf *et al.* 2022) (Fig. 3). Interestingly, mitotic induction by co-expressing ZmMLKS2 and AtCYCD3;1 in tobacco pavement cells visualizes the localization of outer nuclear membrane protein after the envelope breakdown (Xu *et al.* 2020). After mitotic induction, MLKS2 localizes with the preprophase ring in pre-NEBD and around the phragmoplast and new plate region in post-NEBD (Ashraf *et al.* 2022). This study demonstrated the localization of outer nuclear membrane protein during mitosis and corroborated the results of other nuclear membrane proteins’ experiments.

Similar to the outer and inner nuclear envelope proteins, NPC proteins remain in the envelope during interphase. Nuclear pore complex proteins NbRAE1 and RAE1 of *Nicotiana benthamiana* localize in the nuclear envelope prior to mitosis. Interestingly, NbRAE1 co-localizes with microtubules in the PPB, not with cortical microtubules (Lee *et al.* 2009). This result indicates a function of NbRAE1 beyond the nuclear envelope prior to mitosis. During mitosis, NbRAE1 localizes around the mitotic spindle, phragmoplast and new cell plate (Fig. 3). The localization of NbRAE1 during mitosis was confirmed by co-localization with microtubules (Lee *et al.* 2009). This study further confirmed the localization of nuclear envelope proteins, in this case NPC protein, during mitosis and associated with the mitotic apparatus. At this point, the question is whether localizations of nuclear envelope proteins during mitosis are specific or not. For example, NPC protein NUP136 in *Arabidopsis* localizes in the nuclear envelope during the interphase. But, after the envelope breakdown, NUP136 dispersed around the cytoplasmic regions rather than co-localizing with specific mitotic apparatus (Tamura *et al.* 2010). Together these observations from different studies suggest that a subset of nuclear envelope proteins are spatially localized during mitosis or co-localized with distinct components of the mitotic apparatus.

No lamin-related proteins have yet been discovered in plant cells. Instead of containing lamin-related proteins, plant cells possess different sets of proteins, including NMCP (nuclear matrix constituent proteins), CRWN (crowded nuclei), initially named as LINC (little nuclei), KAKU4 (binds to CRWN1) (Ciska and Moreno Díaz de la Espina 2013; Sakamoto and Takagi 2013; Goto *et al.* 2014). Among these lamin-related proteins, both celery NMCP proteins (AgNMCP1 and AgNMCP2) localize similarly at the nuclear periphery (Kimura *et al.* 2010). But their localization differs during mitosis. AgNMCP1 localization is observed on the surface of the segregating chromosome. In contrast to AgNMCP1, AgNMCP2 is prominently distributed in the mitotic spindle and cytoplasmic region of the mitotic spindle (Kimura *et al.* 2010) (Fig. 3). At the same time, AtCRWN1-4 localizes at the nuclear periphery during interphase; but only AtCRWN1 was observed to co-localize with the chromosomes during mitosis (Sakamoto and Takagi 2013) (Fig. 3). These examples beautifully demonstrate that similar nuclear envelope-associated proteins have distinct localization patterns or probable functions during mitosis (Kimura *et al.* 2010; Sakamoto and Takagi 2013).

Together, these examples highlight that inner nuclear membrane (AtSUN1 and AtSUN2), outer nuclear membrane (AtRanGAP1, AtWIP1, AtWIP2a, AtWIT1, ZmMLKS2), NPC (NbRAE1) and lamin-related (AgNMCP1, AgNMCP2, AtCRWN1) proteins co-localize with distinct mitotic apparatuses during mitosis (Xu *et al.* 2007, 2008; Lee *et al.* 2009; Kimura *et al.* 2010; Graumann and Evans 2011; Oda and Fukuda 2011; Sakamoto and Takagi 2013; Ashraf *et al.* 2022) (Table 1).

**Table 1.** Localization of nuclear envelope proteins during mitosis.

Protein	Source organism	Expressing cell	Experiment method	Mitotic localization	Reference
RanGAP1	*Arabidopsis thaliana*	*Arabidopsis thaliana*	Fluorescent tagging	PPB, CDS, cell plate	Rose and Meier (2001); Xu *et al.* (2008)
WIP1	*Arabidopsis thaliana*	*Arabidopsis thaliana*	Fluorescent tagging	Cell plate	Xu *et al.* (2007)
WIP2a	*Arabidopsis thaliana*	*Arabidopsis thaliana*	Fluorescent tagging	Cell plate	Xu *et al.* (2007)
WIT1	*Arabidopsis thaliana*	*Arabidopsis thaliana*	Fluorescent tagging	Cell plate	Zhao *et al.* (2008)
MLKS2	*Zea mays*	Tobacco pavement cell	Fluorescent tagging	PPB, phragmoplast, cell plate	Ashraf *et al.* (2022)
SUN1	*Arabidopsis thaliana*	Tobacco BY-2 cell	Fluorescent tagging	Spindle, phragmoplast, cell plate	Graumann and Evans (2011)
SUN2	*Arabidopsis thaliana*	Tobacco BY-2 cell	Fluorescent tagging	Spindle, phragmoplast, cell plate	Graumann and Evans (2011)
SUN1	*Arabidopsis thaliana*	*Arabidopsis* suspension culture cell	Fluorescent tagging	Spindle, cell plate	Oda and Fukuda (2011)
RAE1	*Nicotiana benthamiana*	Tobacco BY-2 cell	Immunostaining	PPB, spindle, phragmoplast, cell plate	Lee *et al.* (2009)
NMCP1	*Apium graveolens*	*Apium graveolens*	Immunostaining	Segregating chromosome	Kimura *et al.* (2010)
NMCP2	*Apium graveolens*	*Apium graveolens*	Immunostaining	Spindle	Kimura *et al.* (2010)
CRWN1	*Arabidopsis thaliana*	*Arabidopsis thaliana*	Fluorescent tagging	Segregating chromosome	Sakamoto and Takagi (2013)

## Localization Is Equivalent to Function?

In biological systems, localization is usually a good indicator to attribute a function and this is true for the localization of nuclear membrane proteins. Immunostaining and transgene expression are common methods for visualizing the proteins’ localization. Immunostaining is one of the best ways to address the localization of a protein. In this case, the limiting factors are: (i) availability of antibody against the native protein and (ii) specificity of the antibody for the plant proteins. On the other hand, transgene expression can lead to slightly different patterns of localization in different transgenic lines. Additionally, use of strong constitutive promoter can be subjected to silencing of the transgene or mislocalization, if the protein is present ectopically or unnaturally high levels. Previous studies relied on immunostaining, transgene expression and heterologous expression using constitutive promoters for the localization of nuclear membrane proteins. Consequently, the dynamic localization or interaction of nuclear membrane proteins with cytoskeleton is not always conclusive. As a result, future studies are required to confirm these preliminary data by other methods.

A potential reason for a lack of mitotic phenotypes in nuclear envelope mutants is the high gene copy number. Most of the classes of nuclear envelope genes have multiple copies. To date, there is little evidence for alteration of division plane and mitotic activity. For example, knockout of *Arabidopsis* WPP genes causes reduced mitotic activity in the root, which is demonstrated by shorter roots and a phenotype of fewer lateral roots (Patel *et al.* 2004). Virus-induced gene silencing of NPC protein RAE1 in tobacco causes reduced mitotic activity and increased ploidy level. The altered mitotic activity is observed through the smaller leaf phenotype (Lee *et al.* 2009). Mutation in *Arabidopsis* RanGAP, RanGAP1^RNAi^/*rg2-3*, demonstrates shorter root phenotype and disoriented cell files, either due to oblique or stubbed cell wall (Xu *et al.* 2008). Furthermore, it has been found that AtRanGAP1 physically interacts with POK1 to retain the information of CDSs (Xu *et al.* 2008). These studies highlight roles for nuclear envelope proteins during mitosis and/or division plane maintenance—although higher-order mutants may clarify or unveil additional roles.

Recently, outer nuclear membrane-mediated division site determination has been explained in maize. In the outer nuclear membrane protein mutant *mlks2-1*, the nucleus fails to position correctly prior to division. As a result, the future division site marked by the PPB becomes misplaced and the mitotic apparatus follows the initial instruction, which eventually leads to misoriented asymmetric cell division (Ashraf *et al.* 2022). Initially, *mlks2-1* was identified based on an aberrant asymmetric cell division (Gumber *et al.* 2019b). Interestingly, two other maize nuclear envelope mutants were identified in recent years with similar aberrant asymmetric cell division phenotype. Both *mkaku41* and *aladin1* mutants have aberrant subsidiary cell phenotypes during stomatal development in maize (Best *et al.* 2021; McKenna *et al.* 2021). It is an interesting open question whether MKAKU41 and ALADIN1 (NPC protein) regulate future sites similar to MLKS2 or not.

Altogether, it seems that nuclear envelope proteins not only localize with the distinct mitotic apparatus, but they are also involved in cell division either regulating/maintaining the future division site or contributing to mitotic activity.

## Interaction of Nuclear Envelope Proteins With Cytoskeleton

Nuclear movement and mitosis are mediated through the interaction of nuclear envelope protein with actin, microtubules and motor proteins. This information is considered as common knowledge in the field, but most of these findings are based on the observations from non-plant systems. For instance, outer nuclear membrane protein of *Caenorhabditis elegans*, ANC-1, directly binds with filamentous actin (Starr and Han 2002). In fibroblast cells, both outer (nesprin2G) and inner (SUN2) nuclear membrane proteins move along the actin during polarized nuclear movement and create transmembrane actin-associated nuclear lines (Luxton *et al.* 2010). Similar to the interaction with actin, there are several prominent examples regarding the interaction of nuclear envelope proteins with microtubules and motor proteins. For example, outer nuclear membrane protein ZYG-12 in *C. elegans* interacts with centrosomes and binds with motor protein dynein (Malone *et al.* 2003).

Compared to the non-plant system, there are only a few examples about the interaction of nuclear envelope proteins with cytoskeleton. *Zea mays* outer nuclear membrane protein MLKS2 contains armadillo or ARM domain and its interaction with actin, not microtubule, is mediated through ARM domain during the interphase stage (Gumber *et al.* 2019b). After mitotic induction, MLKS2 localizes with microtubule-related structures such as PPB and mitotic apparatus (Ashraf *et al.* 2022). These results indicate that the interaction of nuclear membrane proteins with actin or microtubule is regulated spatially. Future biochemical assays to determine which plant nuclear envelope proteins (and the domains with them) directly bind cytoskeletal elements would help to determine the functional roles of the proteins within the cell.

Movement of the nucleus inside the cell follows the cytoskeletal tracks and this process requires corresponding motor proteins. In *Arabidopsis*, co-immunoprecipitation (Co-IP) experiments using motor protein myosin XI-i identified outer nuclear envelope protein WITs as interacting partners (Tamura *et al.* 2013). The myosin XI-i signal, using YFP-XI-i-Δmotor, is abolished from the nuclear membrane in *wit1 wit2* double mutant in *Arabidopsis* hypocotyl and root (Tamura *et al.* 2013). A similar approach has been used to identify inner nuclear membrane protein interactors in *Z. mays*. Co-IP of *Z. mays* inner nuclear membrane protein SUN2 identified both actin and microtubule as potential interactors (Gumber *et al.* 2019a). However, the *in vitro* and *in vivo* studies are not performed yet for these interactions.

Only a few nuclear envelope proteins, such as AtRanGAP1, ZmMLKS2 and NbRae1, localize with the PPB. This localization pattern of nuclear envelope proteins with PPB microtubules happens prior to the envelope breakdown. It also raises the possibility that nuclear envelope proteins contain microtubule-binding domains. In this context, it has been demonstrated that PPB-localized NbRae1 directly binds with tubulin in the *in vitro* binding assay (Lee *et al.* 2009). Unfortunately, the *in vitro* tubulin binding assay is not performed for other nuclear envelope proteins, more specifically PPB-localized proteins.

Microtubules nucleate from the γ-Tubulin Complexes (γ-TuCs) localized at the nuclear surface. Previously, it has been demonstrated that GIP1 (GAMMA-TUBULIN COMPLEX PROTEIN3/GPC3-INTERACTING PROTEIN1) and GIP2 localize to the nuclear membrane prior to the envelope breakdown, mitotic spindle, phragmoplast, and reappear on the nuclear envelope of daughter cells (Janski *et al.* 2012). Double-knockout mutant *gip1gip2* causes a reduction of CENH3 (CENTROMERIC HISTONE H3) level at centromeres (Batzenschlager *et al.* 2015). Like the *crwn* mutants, *gip1gip2* double mutant also has aberrant nuclear shape (Sakamoto and Takagi 2013; Batzenschlager *et al.* 2015). Altogether, it suggests that nuclear envelope-localized proteins have probable microtubule-binding capacity.

These studies provide some direct and indirect interaction of nuclear envelope proteins with cytoskeleton and motor proteins. But the future experiments are required to validate previous results and identify unknown actin/MT-binding domains of nuclear membrane proteins.

## Evolution of Land Plant Holds the Clue of Nuclear Envelope Proteins’ Function

One of the major hindrances to study functions of nuclear envelope proteins is their high gene copy number. This problem can be solved by generating higher-order mutants; but generating multi-mutants of important gene families cause sometimes embryo lethality. For instance, the double mutant of RanGAPs, *rg1-1 rg2-3*, is a gametophyte lethal in *Arabidopsis* (Xu *et al.* 2008). In this context, the best approach is looking for the related plant species with lower or if possible single copy of corresponding gene families. Alternatively, inducible or cell-type-specific CRISPR-Cas9 knockout may provide the elegant solution.

**Figure 4.**
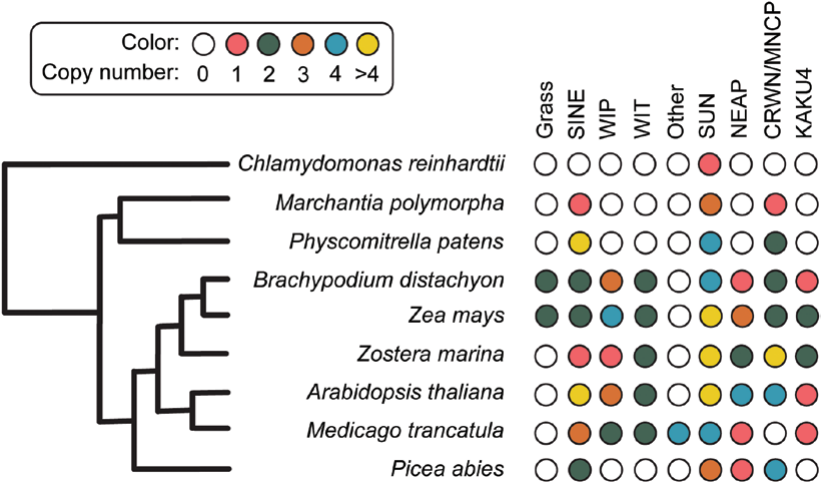
Coulson plot for nuclear envelope proteins in evolutionary diverse land plant species. Gene copy number of distinct classes of nuclear membrane and associated proteins were highlighted for *Chlamydomonas reinhardtii*, *Marchantia polymorpha*, *Physcomitrella patens*, *Brachypodium distachyon*, *Zea mays*, *Zostera marina*, *Arabidopsis thaliana*, *Medicago trancatula* and *Picea abies*. The organism tree is drawn based on the Time Tree of Life (http://www.timetree.org/). Gene copy number, domain organization and protein classification were explained previously by Hank Bass’s group (Gumber *et al.* 2019a).

Land plant evolution provides an excellent opportunity to study the function of nuclear envelope proteins (Gumber *et al.* 2019b; Evans *et al.* 2020). In this part, the focus will be given to the LINC complex and associated proteins. Because LINC complexes and their associated proteins have a characteristic domain organization. They are readily identifiable in other plant species. Considering organisms from the single-cellular *Chlamydomonas reinhardtii* to crop plants like *Z. mays* and the copy number of each nuclear membrane gene families, the majority of these gene families have no copy or low copy number of genes in *C. reinhardtii*, *Marchantia polymorpha*, and *Physcomitrella patens* (Fig. 4). Additionally, two distinct classes are observed only in grass species (*Z. mays* and *Brachypodium distachyon*) or nodule plants (*Medicago trancatula*). On the contrary, we observed no copy and one copy of SINE genes in *C. reinhardtii* and *M. polymorpha*, respectively. But there are seven SINE genes in *P. patens*, which decrease in number in *Arabidopsis thaliana* and *Z. mays*. For a single copy number containing organisms, knocking out the single gene copy will clearly suggest its function through phenotype. Then, complementing the single gene copy knockout lines with higher gene copy number containing organisms one by one will point out the functional role, and redundancy. For instance, NMCP in *M. polymorpha* has a single-copy gene compared to four copy genes of CRWN in *Arabidopsis* (Wang *et al.* 2013, 2021). Additionally, quadruple *crwn* mutant in *Arabidopsis* is lethal, but the single-copy knockout gene of NMCP in *M. polymorpha* is viable (Wang *et al.* 2013, 2021). Another example, AtWIP1 and AtWIP2a localize in the new plate, but AtWIP3 does not localize at the same place (Fig. 3). It indicates the distinct targeting and localization mechanism by AtWIPs, although we do not know the mechanism. Utilizing evolutionary diverse plant specifies will provide insightful information in this context.

Evolution of land plants not only provides distinct gene copy numbers, but also divergence of mitotic apparatus. For instance, *P. patens* do not form characteristic microtubular PPB (Buschmann and Zachgo 2016), Zygnematophyceae lacks phragmoplast (Žárský *et al.* 2022), basal streptophyte algae use microtubules asters and cleavage furrow instead of PPB and phragmoplast, respectively (Buschmann and Zachgo 2016). At the same time, genetic materials are available with double PPB in *cycb1;1 cycb1;2* (Romeiro Motta *et al.* 2022) or PPB-depleted cells in *trm678* mutant (Schaefer *et al.* 2017). Distinct mitotic apparatus-specific localization of nuclear envelope proteins can be explored further for their localization and function using evolutionary diverse organisms and genetic materials.

## Concluding Remarks

Nuclear envelope proteins play dual functions, during interphase and mitosis. Unfortunately, our current knowledge is skewed to their function during the interphase stage. To decipher their function during mitosis, future studies should focus on (i) endogenous localization of nuclear envelope proteins using time-lapse microscopy, (ii) identifying molecular components or actin/MT-binding domain of nuclear envelope proteins and (iii) role of nuclear envelope proteins during the evolution of mitotic apparatus along with land plant evolution. Additionally, advancement in gene editing technology such as CRISPR-Cas9 and super-resolution microscopy techniques will be helpful in future to answer challenging questions in the nuclear envelope protein research.

## Data Availability

No data were generated for this review.

## Notes

**Phenome, Genome & Environment. Chief Editor:** Colleen Doherty
